# The rectum, anal sphincter and puborectalis muscle show different contraction wave forms during prolonged measurement with a simulated feces

**DOI:** 10.1038/s41598-023-50655-7

**Published:** 2024-01-03

**Authors:** Daming Sun, Kar Man Lo, Ssu-Chi Chen, Wing Wa Leung, Cherry Wong, Tony Mak, Simon Ng, Kaori Futaba, Hans Gregersen

**Affiliations:** 1 Chongqing Engineering Research Center of Medical Electronics and Information Technology, Chongqing University of Posts and Telecommunications, Chongqing, China; 2 California Medical Innovations Institute, 11107 Roselle St., San Diego, CA 92121 USA; 3 Department of Surgery, The Chinese University of Hong Kong, Shatin, Hong Kong

**Keywords:** Gastroenterology, Biomedical engineering, Physiology

## Abstract

Contractile patterns in rectum, puborectalis muscle and anal sphincter must be studied to understand defecation. Six subjects had contractile waveforms studied with Fecobionics. Symptom questionnaires, balloon expulsion test and anorectal manometry were done for reference. The Fecobionics bag was filled in rectum to urge-to-defecate volume and measurements were done for 4 h before the subjects attempted to evacuate the device. Pressures and bend angle (BA) variations were analyzed with Fast Fourier Transformation. Four normal subjects exhibited low frequency waves (< 0.06 Hz) for pressures and BA. The waves were uncoordinated between recordings, except for rear and bag pressures. Peak wave amplitudes occurred at 0.02–0.04 Hz. Pressures and the BA differed for peak 1 (p < 0.001) and peak 2 amplitudes (p < 0.005). The front pressure amplitude was bigger than the others (rear and BA, p < 0.05; bag, p < 0.005) for peak 1, and bigger than bag pressure (p < 0.005) and BA (p < 0.05) for peak 2. One subject was considered constipated with lower front pressure amplitudes compared to normal subjects and increased amplitudes for other parameters. The sixth subject was hyperreactive and differed from the other subjects. In conclusion, the rectum, anal sphincter and puborectalis muscle showed different contraction waves during prolonged measurements. The data call for larger studies to better understand normal defecation, feces-withholding patterns, and the implications on anorectal disorders.

## Introduction

The mechanisms of defecation and continence depend on several factors including colorectal motility, stool consistency, rectal capacity and compliance, anorectal sensitivity, and coordination of the pelvic floor muscles and sphincter^1–5^. In this regard, the pressures generated in rectum and by the anal sphincters as well as the anorectal angle, determined by the contractile state of the puborectalis muscle, are important and must be measured in a single integrated test.

Management of patients with functional disorders of the anorectum can be optimized if a better understanding of the multifactorial control of defecation and continence is obtained. Tests for physiological assessment and diagnostics of functional anorectal disorders are available but may not cover all facets of anorectal function or identify the underlying mechanisms. In particular, the opening characteristics of the anal sphincters during incontinent episodes and defecation cannot be described in detail with any currently available exam. Defecography is the only technology that reflects the dynamics of the defecation and quantitates the anorectal angle but unfortunately it does not provide information about anorectal pressures. Furthermore, the balloon expulsion test (BET) assesses the time it takes to defecate the balloon but no other defecatory parameters such as pressure^6,7^, and anorectal manometry (ARM) simulates defecation but does not measure during real evacuation. Disagreement was found between the results of various anorectal tests in some studies whereas others showed higher concordance and that tests are complementary^8–11^. Disagreement may be attributed to differences in technology, measurements, and that they are done at different time points. Furthermore, poor correlation has been found between test results and symptoms^12–16^.

We are seeking to change the approach to anorectal functional testing with the overall goal to provide mechanistic understanding of defecation using a simulated stool named Fecobionics^17–23^. It integrates elements of ARM, BET and defecography. Fecobionics makes it possible to describe the opening characteristics during entry into the relaxing anal canal without disturbing the defecation process. Recently, technological validation^24^ and defecation studies on normal subject and presumed normal subjects with abnormal ARM-BET^17,25^ were published. It was demonstrated that the axial pressure signatures, preload-afterload analysis, and computation of defecation indices (DIs) with Fecobionics provide useful endpoints^17,25^. Studies have also been published on patients with fecal incontinence and obstructed defecation^18,21,23,26^, since these symptoms play a crucial role in the clinic^1,12,15,27,28^. However, only few studies have been published with technology that allows integrated measurements of the various mechanisms controlling continence and defecation. None of the previous Fecobionics studies evaluated contractile patterns during long-term placement in rectum since the insertions merely lasted 20 min or shorter. Intuitively, we expected that rectal contractions will be present due to the urge induced by distension, at least temporarily, but it is not known how the puborectalis and anal sphincter react to sustained rectal balloon distension.

The aim of this feasibility study was to shed light on three elements in the defecatory system with measurement during prolonged filling of rectum, namely the internal anal sphincter tone, the puborectalis tone, and rectal contractility including the frequency specters of the elements and their interrelation.

## Results

Of the six subjects studied, one could not evacuate BET within two minutes and was considered abnormal (consistent with defecatory disorder, which includes obstructed defecation or dyssynergia). Another subject had prolonged periods of sustained rectum contractions. This was also considered abnormal; i.e., as a case of rectal hyperreactivity. The bag volume was within the normal range (80 mL), i.e., the subject was not hypersensitive. Both are described below as individual abnormal cases. This left us with four subjects that we considered normal, i.e., they had normal FISI and constipation scores, normal ARM-BET data and evacuated Fecobionics within 2 min (17–96 s). These subjects are further characterized as the normal group below.

### Normal group

The four subjects were all adult men aged 23.8 ± 1.9 years, height 169.9 ± 4.7 cm and weight 60.9 ± 1.6 kg. The urge volumes were 10, 40, 45 and 80 mL, respectively. All subjects kept the device in rectum for 4 h after bag filling. The subjects felt urge only for a short time (less than 15 min) and the urge sensation did not return during the 4 h period. Two subjects were in seated position during the whole study whereas the two others had one shift between sitting and laying.

**Figure 1:**
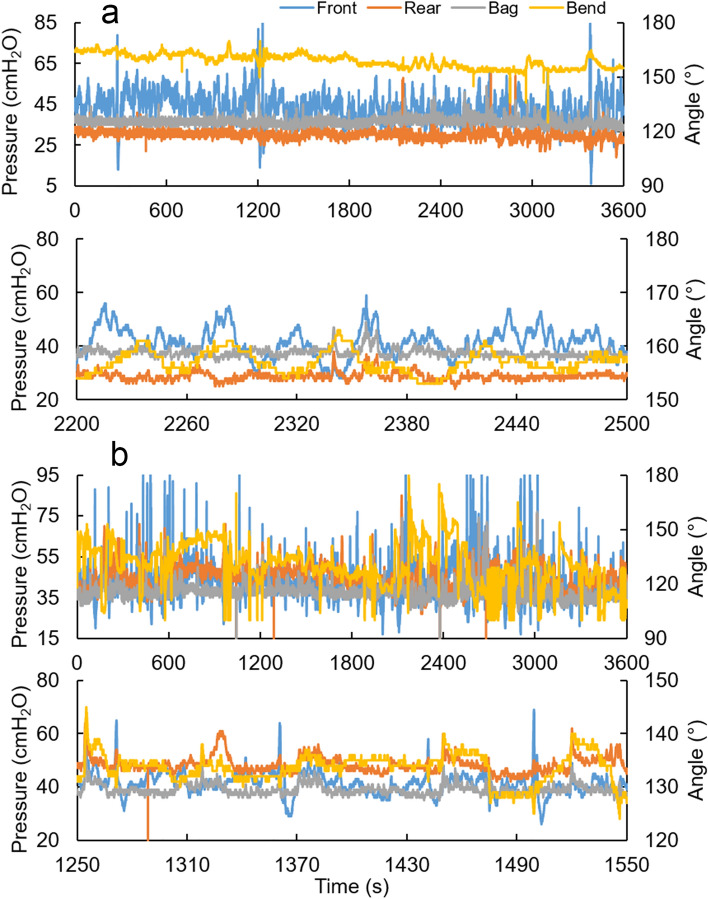
Representative recordings during deferred defecation. (**a,b**) Upper panels show continuous recordings of 1 h from two subjects. The lower panels for (**a,b**) show a representative 5 min periods from the 1-h recording. Notice that the scales differ between the graphs. Largely independent contractions were observed in the pressure and bend angle recordings. Two contractile wave forms were observed in each pressure and bend angle. One was a slow wave (tone) that repeated itself every 40–50 s. Imposed on the slow waves, the other represented faster (phasic) contractions imposed on the slow contraction waves.

Figure 1 shows representative pressure and bend angle recordings from 1 h continuous recordings and a 5-min period from within the 1 h recording from two of the subjects (not close in time to change in posture or movement of the subject, which caused some random variation, especially on the bend angle). Contraction-relaxation waves were observed for the pressures and bend angle. Subject A in Fig. 1 shows prominent front pressure waves (anal sphincter contraction and relaxation) whereas subject B shows a more random pattern. One wave form was a slow wave that repeated itself every 40–50 s (around 0.02 Hz). Imposed on the slow waves, we observed faster contractions with frequency around 0.1 Hz and amplitudes around 5–10 cmH_2_O. Because the faster wave form was variable for various reasons, such as breathing, we focus on the slow wave in this paper.

In the selected 5-min periods, the average front, rear, and bag pressures were 36.5 ± 1.9, 32.4 ± 1.5 and 34.6 ± 1.1 cmH_2_O, respectively. The bend angle was 137.7 ± 5.4 degree. No significant difference was found for each parameter between the time periods, i.e., from the beginning to the end (ANOVA, front: F = 0.154, p = 0.154; rear: F = 0.571, p = 0.578; bag: F = 0.524, p = 0.602; bend: F = 0.464, p = 0.637). Visually inspecting the recordings, we found that the waves among the four recordings were relatively uncoordinated, e.g., the pressure waves in one recording would often not be simultaneous or associated with waves in the other pressure recordings or in the bend angle. This was confirmed by spectrum analysis of the subtracted values between signals, where we found stochastic frequency components with a large range. This was, however, much less for the subtracted values of the rear and bag pressure, demonstrating that these two recordings basically measure the same contractile events.

**Figure 2:**
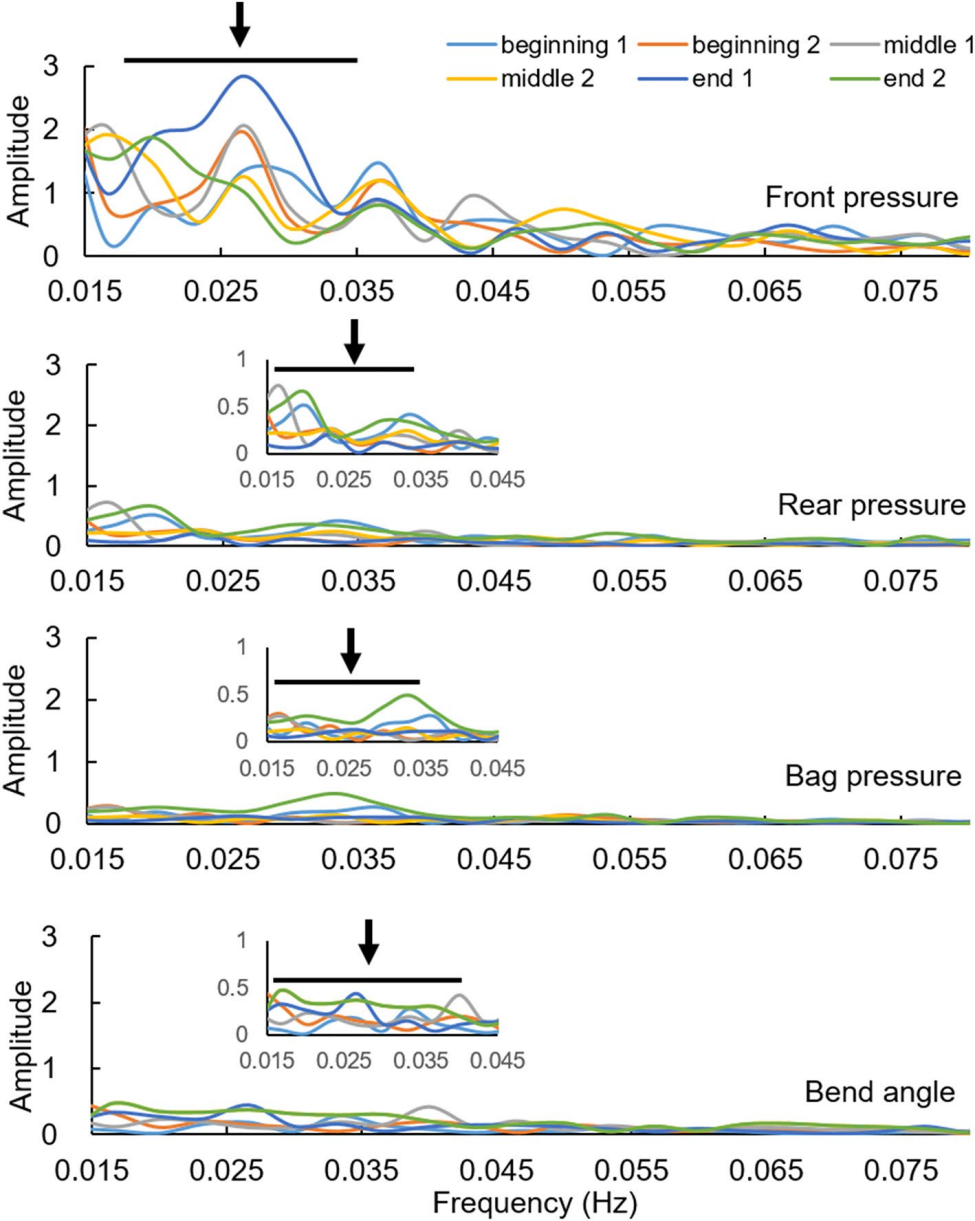
Typical frequency domain graphs of one period. Low frequency signals were found in all parameters after low-pass filter. The distinct amplitude locates around 0.02–0.04 Hz, and it became very small after 0.06 Hz. (arrow: the range of peaks with more than 30% deviation from the baseline).

The frequency domain result is illustrated in Fig. 2 for a representative subject. Low frequency signals (< 0.06 Hz) were found in each pressure and for the bend angle. The peaks with biggest amplitude were located around 0.02–0.04 Hz. Outside this range, the peaks were small. There were no significant differences of frequency and amplitude of the two biggest peaks during the time course for each parameter in this subject (p > 0.1).

**Figure 3:**
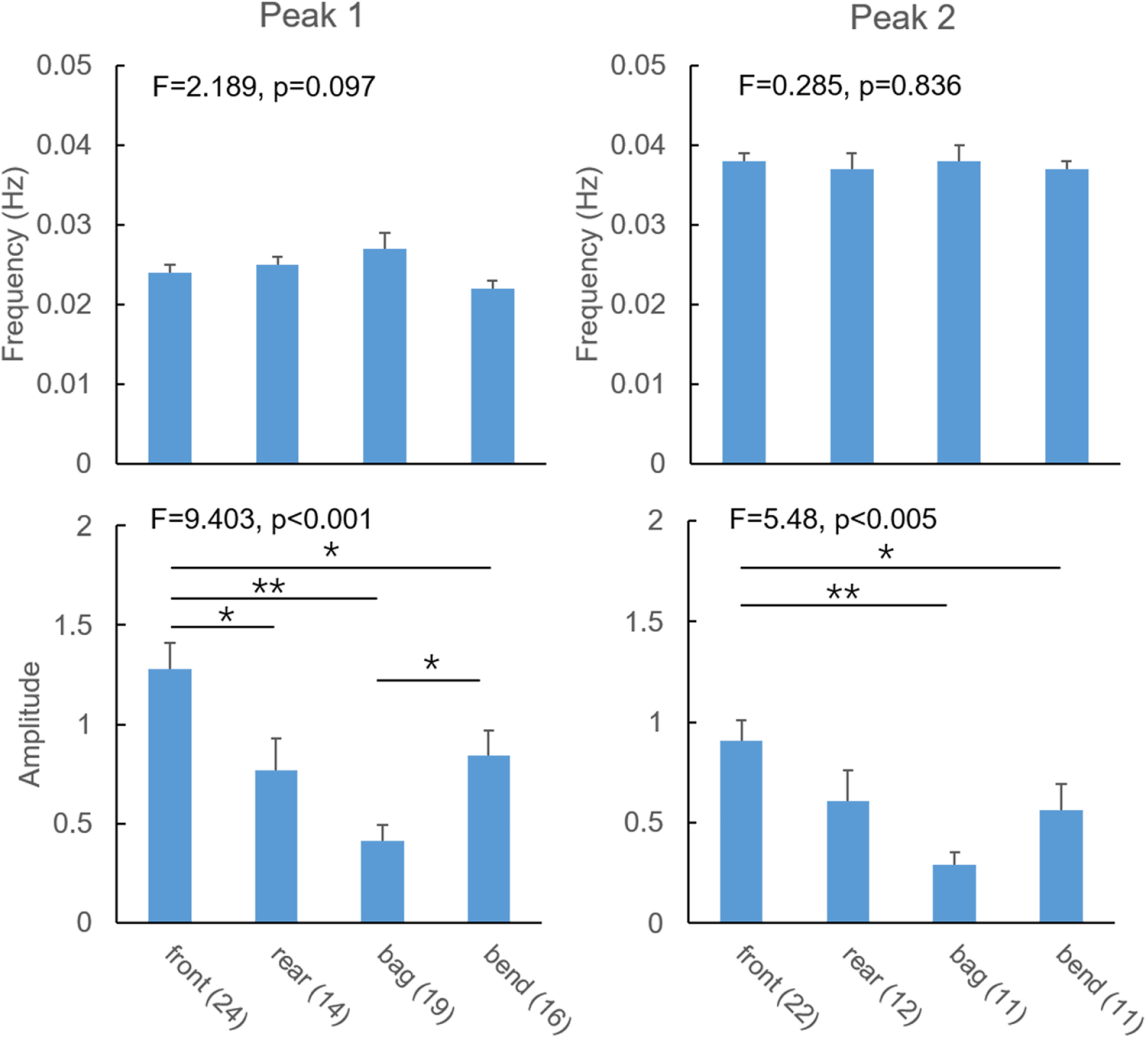
The frequency peaks and amplitudes for the pressures and bend angle. Overall, the amplitudes differed between the four recordings. The stars * indicate significant difference. *p < 0.05 and **p < 0.005.

Figure 3 shows the frequency peak and amplitude differences for the pressures and bend angle for the normal group. Peaks according to the criteria described in the Methods were identified for all six 5-min periods in all normal subjects for the front pressure, except for two cases of peak 2. However, peak 1 and 2 were less frequently identifiable for the other recordings, i.e., rear pressure 58% and 50%, bag pressure 79% and 46%, and bend angle 67% and 46% for peak 1 and 2, respectively. The frequency of peak 1 did not differ between the four parameters. The same was the case for peak 2 (Fig. 3). Significant differences were found between each parameter on amplitude (Peak 1: F = 9.403, p < 0.001; Peak 2: F = 5.48, p < 0.005). The post hoc test showed that the overall difference was due to the front pressure amplitude being significantly bigger than the others (rear and bend, p < 0.05; bag, p < 0.005) for peak 1, and significantly bigger than the bag pressure (p < 0.005) and bend angle (p < 0.05) for peak 2.

### Case with constipated subject

One subject, a 48-year-old female, who could not evacuate BET within 2 min was characterized as a being constipated though her constipation score was normal. The filling volume to urge was 60 ml, and she kept the device in rectum for 4 h after bag filling, i.e., deferred defecation for 4 h without sensing urge again. This subject had one shift between sitting and laying. She did not evacuate Fecobionics within 2 min.

**Figure 4:**
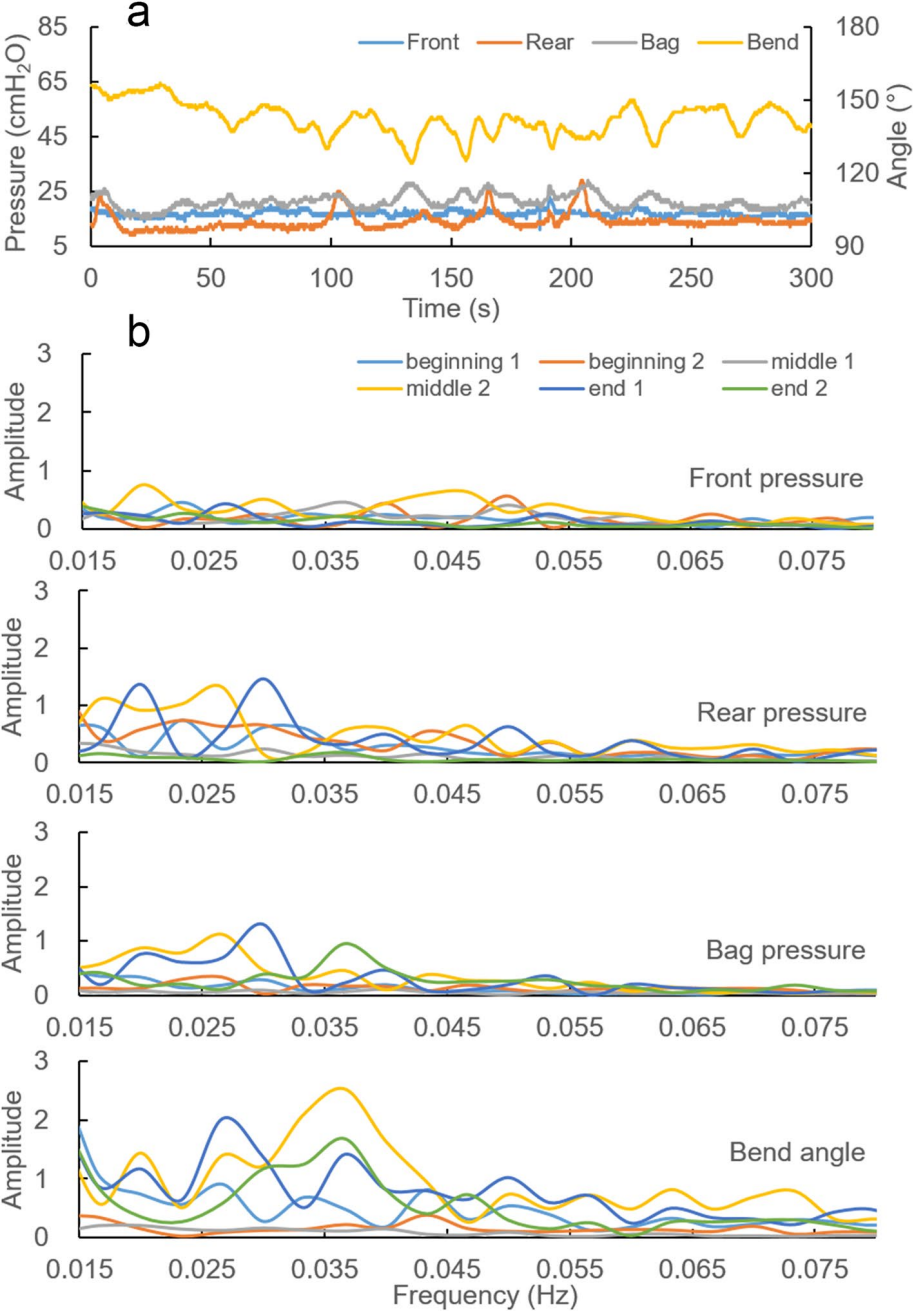
Data from the subject who was considered constipated due to prolonged expulsion time of BET and Fecobionics. (**a**) A typical 5-min period recording. Largely independent contractions were observed in the four recordings. There is no clear slow wave in front pressure compared to what we observed in the normal subjects. (**b**) Frequency domain graphs of each parameter for the six 5 min periods for the constipated subject. There was no distinct peak in front pressure wave. The amplitudes of the other parameters have big frequency domain overlap.

Figure 4a shows a typical 5-min recording period for the constipated subject. The subject had distinct bend angle waves and these waves were often associated with simultaneous pressures changes. Slow waves in the front pressure were not observed.

In the selected 5-min periods, the front, rear, bag rest pressures were 24.5 ± 3.7, 20.9 ± 5.3 and 26.7 ± 3.2 cmH_2_O, respectively. The bend angle was 134.8° ± 6.0°. Frequency domain graphs of each parameter for the selected periods are shown in Fig. 4b. Low frequency signals were found for all pressure and bend angle parameters. However, they appeared different from the normal group. There were more compositional waves in the frequency domain. The waves with high amplitude were also located around 0.02–0.04 Hz but the frequencies reached 0.08 Hz. Furthermore, the front pressure wave amplitudes were lower than in the normal group, and often no distinct peaks could be found. The amplitudes of the other three parameters increased compared to the normal group. A large frequency domain overlap was found.

### Case with hyperreactive subject

**Figure 5:**
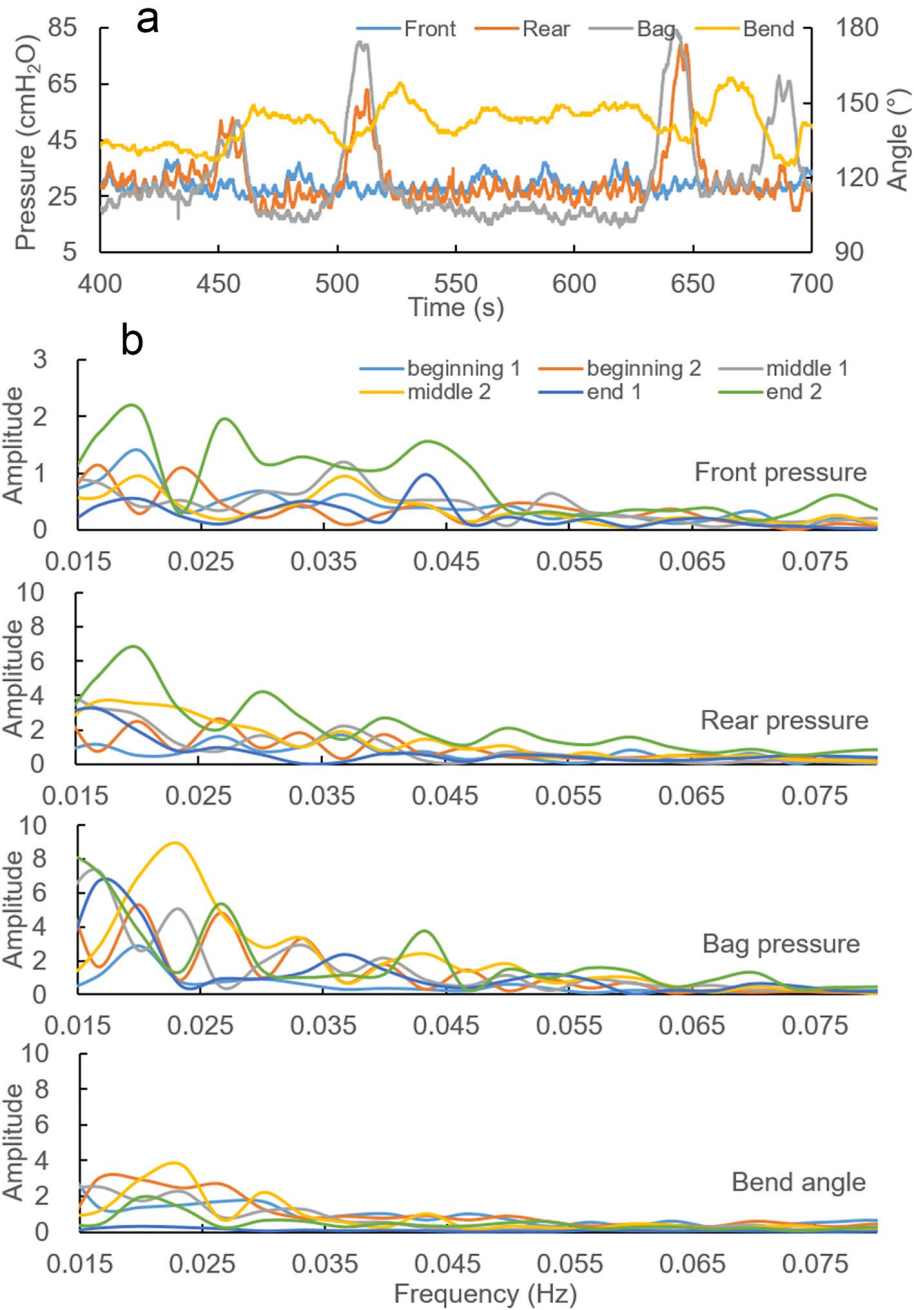
Data from the subject who was considered hyperreactive due to the vigorous contractions in the rear channel (**a**) A typical 5-min period recording. Random changes of rear and bag pressures occur following bend angle variations. (**b**) The frequency domain graphs of each parameter for the six 5-min periods for the hyperreactive subject. Component waves with big amplitude occurred in the bag and rear pressures and in the bend angle. The frequency peak of the front pressure shifted to the right compared to the normal subjects.

One subject, a 21-year-old man had an urge volume of 80 ml but exhibited contractions to an extend that was not observed in other subjects. After the four hours measurements, he evacuated the device in 32 s. He had normal questionnaire scores. BET and ARM studies were normal. This subject had one shift between sitting and laying. A typical 5-min period recording of this subject is shown in Fig. 5a. Complex wave forms representing contractions and relaxations were observed. The contractions were subtle at low distension volumes and became pronounced during the measurement period One contraction type was random big alterations in bag and rear pressure and in the bend angle. Another type was a faster wave with frequency around 0.1 Hz in all pressure channels.

The frequency domain graphs are shown in Fig. 5b. Low frequency signals were found for all parameters as in the normal group. Component waves with particularly large amplitudes were located between 0.02 and 0.03 Hz for the bag and rear pressure. The bend angle had similar characteristics but with smaller amplitude. Furthermore, the front pressure frequency peak appeared right shifted, and the amplitude was the same as in the normal group, i.e., the biggest peak frequency increased to upper edge of 0.2–0.4 Hz and close to 0.5 Hz.

## Discussion

The small-scale feasibility study showed slow contraction-relaxation waves for the rectum, puborectalis muscle and anal sphincter during prolonged recordings with the subjects deferring defecation. The basic frequencies were similar between the three anatomical regions, but the amplitudes varied considerably. We noticed rigorous waves in the front channel recording from the anal sphincter. Spectrum analysis demonstrated lack of coordination between the pressures and bend angle recordings, except for the rear and bag pressure. The two subjects that we characterized as abnormal, had distinctly altered patterns compared to the normal group.

### Methodological aspects and limitations

Low correlation has been documented between various anorectal function tests and between tests and symptoms, which impedes detailed analysis of defecatory properties. This may be due to that the tests are done on separate occasions, i.e., are not integrated. A single integrated test that measures relevant physiological and pathophysiological parameters is warranted for obtaining a better understanding of defecation and continence mechanisms. This study used a novel simulated stool named Fecobionics to describe and analyze the contributions from the internal anal sphincter, the puborectalis muscle, and rectum including the frequency specters of these elements and their interrelation during prolonged filling of rectum.

The major limitation is the size of the material with only six subjects included, and two subjects appeared to be abnormal. The abnormality was defined by the Fecobionics testing rather than by symptom scores and by testing with current gold standard technology though the subject who could not expel the Fecobionics device also did not expel the BET balloon. Furthermore, the urge-to-defecate volumes were quite variable, even in the group we defined as normal. The above may constitute an immediate problem, on the other hand, anorectal physiology is highly variable, and current tests often show overlap between normal subjects and patients with defecatory disorder, e.g., ARM shows paradoxical contraction, a sign of dyssynergia, in 85–90% of normal subjects and some patients can expel the BET balloon within a period considered normal^1,3,4,8^. The small size of the material limits wider conclusions and definition of normal ranges, as well as gender and age analysis. Gender and age must be considered in larger follow-up studies. On the other hand, the recordings of waves from the four normal subjects were quite identical, and the two abnormal subjects differed in contractile phenotypes. During the experiments, we observed bend angle variation when the subjects changed position. Position changes should ideally be avoided in future studies. Furthermore, faster waves are more likely influenced by excursions caused by respiration and movements. Therefore, we used low pass filtering before the FFT. This process decreased the amplitude of each parameter, i.e., the real signals have bigger amplitudes.

It is a matter of interest if feces move retrograde in the intestines, i.e. proximal of rectum if defecation is deferred. However, we did not find significant changes in the wave forms and especially in the bend angle between the time periods of the study. Hence, we believe that the device stayed in rectum until the defecation attempt.

### Physiological aspects

Many adults and children are forced to defer defecation during daily activities such as being in school classes or meetings. By deferring defecation, human subjects control defecation or flatus until social conditions are appropriate. This is done by suppressing the urge sensation, as well as controlling the contractile state of the puborectalis muscle and external anal sphincter. Other elements in the defecatory system such as rectal contractility and relaxation of the internal anal sphincter are not under voluntary control^29–32^. Deferred defecation is considered a cause of chronic constipation^27,28,33^, but it may be associated with other anorectal problems as well. Prolonged stool retention in the rectum may result in increased anorectal volumes with failure of contraction of the external anal sphincter, resulting in soiling^27^. It is likely that associated delay in rectosigmoid transit time leads to secondary prolongation of colonic transit time, i.e., slow transit constipation^34,35^. The excess stool accumulated in the rectum may lead to dilated rectum and hyposensitivity to rectal filling^36^. Our study design constitutes a potential model of deferred defecation since measurements with the bag filled to urge-to-defecate level were done for 4 h.

Anal pressure waves have been recorded with ARM^37^, and the frequency seem consistent with that measured by the front pressure sensor in this study. To the best of our knowledge, no studies had been published on the coordination between sphincter, puborectalis and rectal contractions during prolonged measurements due to lack of integrated measurement technologies. The contractions in anorectum are usually regarded as a unit without consideration of the different neuromuscular regulation. According to frequency domain analysis, the frequency components of the signals with big amplitudes are with similar trend, i.e., low frequency components around 0.02–0.04 Hz. This is also the case for the pressures and bend angle in this study. The waves recorded by the pressures and bend angle measurement reflect the contractility of the elements of the defecatory system, i.e., the rectum, puborectalis muscles and anal sphincter^38,39^. If two simultaneous recordings have relatively fixed difference, the frequency spectrum of one recording subtracted from another, e.g. the front pressure subtracted from the rear pressure, should show relatively deterministic frequency components. However, we found that the waves recorded in the different sensors were uncoordinated, except for the rear and bag pressures. The front pressure measures the activity of the internal anal sphincter, the bend angle reflects the puborectalis activity and the bag and rear pressures represent rectal contractility^1,3,4^. The major differences were found for the contraction energy, i.e., for the amplitudes (Fig. 3). The amplitude can be regarded as the energy, e.g., the energy is higher for the front pressure (internal anal sphincter waves) than for the other recordings during the prolonged recordings. Hence, abnormal recordings and symptoms can be considered in two ways; the disorders related to contraction coordination and to energy. The inability to generate an adequate propulsive force synchronized with relaxation of the puborectalis and the external anal sphincter is an important characteristic of constipation^11,40^. We found more harmonic waves in the constipated case than in the normal group. This is evidence for a frequency disorder. Furthermore, the amplitude of the front pressure was diminished but the others were increased. The rectal and puborectalis contractions may be more active in the deferment of constipation. In this study, we also had a case with rectal hyperreactivity with excess rectal contractions. The hyperreactive case showed more frequent rectal contractions with high energy. Pronounced phasic activity and high amplitude contraction frequency are features of rectal hypersensitivity^41^, though the distension volume in this subject did not point towards hypersensitivity. The right shift of the front pressure frequency peak indicates faster anal sphincter contraction/relaxation waves. The particularly larger amplitude component waves indicate abnormal high energy for rectal and puborectalis contractions.

## Conclusions

The feasibility study showed slow contraction-relaxation waves for the rectum, puborectalis muscle and anal sphincter during prolonged recordings with the subjects deferring defecation. The waves were most rigorous in the front pressure recording, indicating cyclic pressure changes in the anal sphincter during prolonged measurements. The basic frequencies were similar between the three anatomical regions, but the amplitudes varied considerably, and the activities were not coordinated except for the rear and bag pressures. The two abnormal subjects had altered patterns compared to the normal group. The data call for larger studies to better understand the implications on anorectal disorders.

## Materials and methods

### Subjects

Six normal subjects were invited to participate in this exploratory study in the anorectal physiology lab in the Department of Surgery at Prince of Wales Hospital in Hong Kong. Recruitment was made by local advertisement or by contacting subjects who had previously been participating in our anorectal experiments as normal subjects. The inclusion criterion was asymptomatic normal persons aged over 18 years who gave informed consent. No upper age limit was imposed. The exclusion criteria were persons with history of chronic constipation or FI, abdominal pain, prior abdominal, pelvic and anal surgery, medication and diseases that may affect bowel function and defecation such as cancer, diabetes and infectious diseases. Data were obtained on age, health status, symptoms, diseases, and previous treatments. FI Severity Index (FISI), FI QOL, and constipation scores were obtained^42–44^. The subjects had ARM-BET done at the same day if recordings were not available from other studies within the past three months. The subjects were recruited from June to December 2021.

Prior to functional testing, the subjects were asked to empty their rectum if they were able to. Enema was not used to make the test as natural as possible. Digital rectal examination was performed prior to insertion of Fecobionics to assess anal tone and verify that the lower rectum was empty. Experiments using Fecobionics and ARM-BET were done in randomized order if done on the same day using a predefined scheme with at least 20 min between the tests. The London protocol for ARM was followed. All subjects had the tests completed. FISI scores < 5 was considered normal^43^ and constipation scores below 8 were considered normal^45^. The study was IRB approved (protocol no. 2017.122, Joint CUHK-NT East Cluster Clinical Research Ethics Committee). All experiments were performed in accordance with relevant guidelines and regulations. Trial Registration. https://www.clinicaltrials.gov Identifier: NCT03317938. Date of registration: 23/10/2017.

### Fecobionics

**Figure 6:**
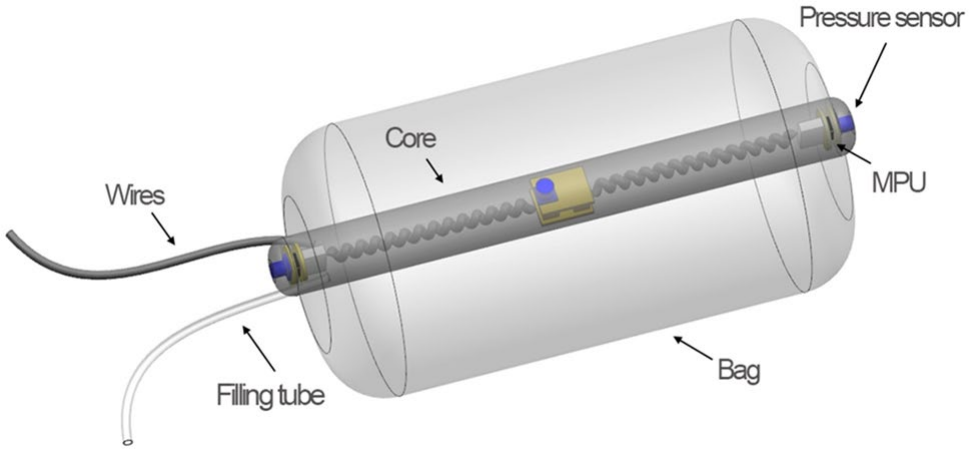
Sketch of the Fecobionics probe used in the studies. The core is made from soft silicone resin and contains three pressure sensors placed at the front, rear and inside the bag. Two 9-axis motion processor units (MPUs) were imbedded for determination of orientation and bend angle during defecation. A filling tube was attached to the front for filling the bag. A cable was attached for data transmission and power supply.

The basic design of Fecobionics has been described (Fig. 6) ^24,25^. Fecobionics was 10-mm-OD, 10-cm-long and made of Silicone rubber (PS6600, Yipin Mould Material Ltd, China). It contained pressure sensors and electronic circuit boards. Miniature pressure sensors (MS5837-30BA, TE connectivity, USA) were embedded in the silicone rubber core at the front, inside the bag, and at the rear of the core. The front and rear sensors pointed in the direction of the defecatory trajectory. Two 9-axis Motion Processor Units (MPUs) (MPU9250, InvenSense, USA) for orientation, angle, and bending measurements were placed towards the front and rear of the probe.

A 30 μm-thick and 8 cm-long polyester-urethane bag spanned most of the core length. The spherically shaped bag contained up to 80 mL without being stretched and had a maximum diameter of 5 cm. The bag was connected through a thin tube extending from the front of Fecobionics to a syringe containing saline.

With the architecture, silicone hardness shore (A5) and the bag, Fecobionics obtained shape and consistency that corresponds approximately to type 4 (range 3–4) on the Bristol stool form scale^46^. The range from types 3–4 is found in + 60% of normal subjects^43^. Wires were threaded inside a thin tube extending from the front to a PCs USB port for power supply and real-time data transmission and display of data. Further processing was done in Matlab.

### Procedures

The settings were made as private as possible using a curtain to shield the patient. Fecobionics was manually inserted into the rectum. Based on previous studies, the rear, bag, and front pressure channels record from the proximal rectum, mid-rectum, and distal rectum touching the proximal anal sphincter, respectively^17,18,21^. After checking probe placement by asking the subjects to squeeze the anal sphincter and coughing, the bag was filled with liquid until the subjects felt the urge to defecate. The urge volume was noted. The subjects were asked to defer defecation for 4 h. They were allowed to sit on a chair or lay on the hospital bed. Changes in posture were recorded. They were asked to rest and refrain from contracting the abdominal muscle unnecessarily. After measurements for 4 h, the subjects were asked to evacuate Fecobionics as they normally do at home and without excessive straining. The investigators left the room, and the subjects defecated the device in privacy.

The Fecobionics devices were inspected for leaks and damage or malfunction. Any safety issue and adverse effects were characterized and reported as unanticipated adverse device effects. No adverse effects were recorded. The subjects were instructed to contact a specific member of the research team if they experienced any problem after leaving the clinic.

ARM-BET was conducted with a standard HRM single-use 8ch anorectal catheter (G-90150, MMS, Enschede, Netherlands). It was inserted with the subjects lying in left lateral position with bended hip and knees. The bag was placed in the rectum and pressure was measured at 0.5 cm distance in the anal canal. Resting anal pressure, maximum anal squeeze pressure, the recto-anal inhibitory reflex (RAIR), urge volume, maximum tolerable volume, and expulsion duration for the 50 mL balloon were evaluated. BET was done on the commode chair and the investigators left the room during BET defecation.

### Data analysis

Pressures were recorded directly by the pressure sensors with atmospheric pressure as zero. Data from the MPUs were more complex and required processing to compute the bend angle of the device. The Madgwick algorithm is an orientation filter applicable to MPUs consisting of tri-axial gyroscopes, accelerometers and magnetometers^47^. An estimated orientation of the sensor frame relative the earth frame, ${q}_{est,t}$, is obtained through the weighted fusion of the orientation calculation, ${q}_{\omega ,t}$ and ${q}_{\nabla ,t}$ with a simple complementary filter:

1 $${q}_{est,t}={\alpha }_{1}{q}_{\nabla ,t}+\left(1-{\alpha }_{1}\right){q}_{\omega ,t}, 0\le {\alpha }_{1}\le 1$$

where ${\alpha }_{1}$ and $(1-{\alpha }_{1})$ is the weights, ranging between 0 and 1, applied to each orientation calculation. In this study, it was used to estimate the accurate orientation of each MPUs, ${q}_{est,t}^{front}$ and ${q}_{est,t}^{rear}$. The raw $\upphi$, pitch $\uptheta$ and yaw $\uppsi$ is calculated as:

2 $$\left[\begin{array}{c}\phi \\ \theta \\ \psi \end{array}\right]=\left[\begin{array}{c}atan2(2\left({q}_{0}{q}_{1}+{q}_{2}{q}_{3}\right), 1-2\left({q}_{1}^{2}+{q}_{2}^{2}\right))\\ {\text{asin}}(2\left({q}_{0}{q}_{2}-{q}_{3}{q}_{1}\right))\\ atan2(2({q}_{0}{q}_{3}+{q}_{1}{q}_{2}, 1-2\left({q}_{2}^{2}+{q}_{3}^{2}\right))\end{array}\right]$$

where ${q}_{0}, {q}_{1}, {q}_{2}, {q}_{3}$ is the quaternions of ${q}_{est,t}^{front}$ and ${q}_{est,t}^{rear}$. From multiple experiments, we found that the bend angle was more related to the pitch angle, i.e., it was defined as:

3 $$bend=\{\begin{array}{c}180^\circ -\left|\left|{\uptheta }_{{\text{rear}}}\right|-\left|{\uptheta }_{{\text{front}}}\right|\right|, \left|\left|{\uptheta }_{{\text{rear}}}\right|-\left|{\uptheta }_{{\text{front}}}\right|\right|, <{90}^{^\circ }\\ \left|\left|{\uptheta }_{{\text{rear}}}\right|-\left|{\uptheta }_{{\text{front}}}\right|\right|, \left|\left|{\uptheta }_{{\text{rear}}}\right|-\left|{\uptheta }_{{\text{front}}}\right|\right|, \ge 90^\circ \end{array}$$

180° means that the probe is straight.

The entire recordings were viewed after the studies to get a visual impression of the collected data. For further analysis, we picked six 5-min time periods, when we had stable signals in the beginning, middle (after 2 h), and at the end of the 4 h measurement period, i.e., two periods from each part of the study.

Fourier analysis converts a signal from its original domain to a representation in the frequency domain and vice versa. The discrete Fourier transform (DFT) is obtained by decomposing a sequence of values into components of different frequencies^48^. This operation is useful in many fields, but computing it directly is often too slow to be practical. A Fast Fourier Transform (FFT) rapidly computes such transformations by factorizing the DFT matrix into a product of sparse (mostly zero) factors^49^. In this study, FFT was used for frequency domain analysis on the pressure and bend angle recordings. In order to filter out high-frequency clutter, all data were low-pass filtered. The frequency peaks were defined as the highest point of each phase of amplitude rise with more than 30% deviation from the preceding baseline. The frequency and amplitude of the first two major peaks were analyzed. Furthermore, the distribution characteristics of the frequency components of subtracted recordings, e.g., the front pressure subtracted from the rear pressure, was used to evaluate their coordination.

### Statistics

Data were expressed as mean ± SEM unless otherwise stated. One-way ANOVA was used for statistical testing of parameters during the time course of the 4 h measurement period. Least Significance Difference (LSD) was used in post hoc test to compare each parameter (IBM SPP Statistics 22, IBM Corp.). The results were considered significant when p < 0.05.

## Data Availability

Access to data can be granted upon reasonable request, which should be directed to the corresponding author.
